# Validation of an International Classification of Disease, 10th revision coding adaptation for the Charlson Comorbidity Index in United States healthcare claims data

**DOI:** 10.1002/pds.5204

**Published:** 2021-03-04

**Authors:** Julie Beyrer, Janna Manjelievskaia, Machaon Bonafede, Gregory Lenhart, Sandra Nolot, Diane Haldane, Joseph Johnston

**Affiliations:** ^1^ Global Patient Outcomes and Real World Evidence Eli Lilly and Company Indianapolis Indiana USA; ^2^ IBM's Life Sciences IBM Watson Health Cambridge Massachusetts USA

**Keywords:** administrative claims, Charlson Comorbidity Index, comorbidity, ICD‐10, United States, validation

## Abstract

**Purpose:**

An International Classification of Disease (ICD‐10) Charlson Comorbidity Index (CCI) adaptation had not been previously developed and validated for United States (US) healthcare claims data. Many researchers use the Canadian adaption by Quan et al (2005), not validated in US data. We sought to evaluate the predictive validity of a US ICD‐10 CCI adaptation in US claims and compare it with the Canadian standard.

**Methods:**

Diverse patient cohorts (rheumatoid arthritis, hip/knee replacement, lumbar spine surgery, acute myocardial infarction [AMI], stroke, pneumonia) in the IBM® MarketScan® Research Databases were linked with the IBM MarketScan Mortality file. Predictive performance was measured using c‐statistics for binary outcomes (1‐year and postoperative mortality, in‐hospital complications) and root mean square prediction error (RMSE) for continuous outcomes (1‐year all‐cause medical costs, index hospitalization costs, length of stay [LOS]), after adjusting for age and sex. C‐statistics were compared by the method of DeLong and colleagues (1988); RMSEs, by resampling.

**Results:**

C‐statistics were generally high (≥ ~ 0.8) for mortality but lower for in‐hospital complications (~0.6–0.7). RMSEs for costs and hospitalization LOS were relatively large and comparable to standard deviations. Results were similar overall between the US and Canadian adaptations, with relative differences typically <1%.

**Conclusions:**

This US‐based coding adaptation and a previously published Canadian adaptation resulted in similar predictive ability for all outcomes evaluated but may have different construct validity (not evaluated in our study). We recommend using adaptations specific to the country of data origin based on good research practice.

**KEY POINTS**

- The Charlson Comorbidity Index (CCI) is a widely used algorithm for measuring comorbidities and risk adjustment, but an International Classification of Disease (ICD‐10) adaption of the CCI for United States (US) healthcare claims data sources had previously not been developed and validated.
- We developed and evaluated the predictive performance of an ICD‐10 US adaptation of the CCI and compared its performance with an ICD‐10 Canadian adaptation (Quan et al 2005) for outcomes of 1‐year mortality, postoperative mortality, in‐hospital complications, 1‐year medical costs, index hospitalization costs, and index hospitalization length of stay.
- C‐statistics for mortality outcomes using either the US or Canadian adaptation were close to or exceeded 0.8 for patient cohorts (range 0.670–0.875), indicating high predictive power for 1‐year and postoperative mortality.
- Predictive power was equally poor for both CCI coding adaptations for in‐hospital complications, 1‐year medical costs, index hospitalization costs, and index hospitalization length of stay.
- Using the Canadian adaptation had minimal impact on predictive ability but could result in different construct validity (i.e., erroneous assignment of individual comorbidities) given different ICD‐10 coding adaptations and real world reimbursement environments in Canada and the US; construct validity was not evaluated in our study. We recommend using adaptations specific to the country of origin of the data, consistent with good research practice guidelines.

## 1 INTRODUCTION

Comorbid illnesses are important indicators of health, and their presence increases the risk of mortality and other health outcomes. The Charlson Comorbidity Index (CCI) was developed to measure the burden of disease from comorbidities and predict 1‐year mortality risk.^1^ The CCI is a commonly used algorithm to measure comorbidities and adjust for risk in health outcomes and similar studies; a Google Scholar search for the published CCI validation^1^ resulted in more than 30 000 citations. The CCI score is determined by 19 comorbid conditions, and a weight is assigned to each based on its severity. The weights for an individual are summed to derive his or her CCI score. With the widespread use of administrative databases for clinical outcomes research, adaptations of the CCI to the International Classification of Disease, 9th Revision, Clinical Modification (CM) were created and validated.^2^, ^3^, ^4^, ^5^, ^6^, ^7^, ^8^, ^9^, ^10^

In 1992, the tenth revision to the International Classification of Disease (ICD‐10) was published by the World Health Organization,^11^ and efforts were made outside the United States (US) to adapt the CCI to ICD‐10.^12^, ^13^, ^14^ Sundararajan et al (2007)^15^ compared the performance of three ICD‐10 coding adaptations and reported that the Canadian version by Quan et al^14^ outperformed the others.^12^, ^13^ Discrepancies among the adaptations may have arisen due to different approaches used to develop them, as well as variations among country‐specific ICD‐10 modifications. The performance of these CCI adaptations in US healthcare data is unknown because different ICD‐10 coding adaptations and realworld reimbursement requirements of different countries can influence the performance of algorithms.^16^ We developed an ICD‐10 coding adaptation for the US^17^; the full current code set is available in machine‐readable files at https://doi.org/10.5281/zenodo.3604394. The primary objective of this study was to validate our ICD‐10 CCI adaptation in a US administrative claims database that included mortality data. We evaluated the predictive performance of our ICD‐10 CCI coding adaptation and compared its performance with that of the ICD‐10 CCI coding adaptation developed in Canada,^14^ which is currently being used by many for US healthcare research since there is presently no published validated algorithm for US healthcare claims.^18^, ^19^, ^20^ Rather than focusing on a single condition or outcome, we sought to validate the coding adaptation for diverse cohorts (i.e., rheumatoid arthritis, hip or knee replacement, lumbar spine surgery, acute myocardial infarction [AMI], stroke, and pneumonia) and outcomes (i.e., 1‐year and postoperative mortality, in‐hospital complications, 1‐year all‐cause medical costs, index hospitalization costs, and length of stay [LOS]).

## 2 METHODS

### 2.1 Coding adaptations

The same comorbidities and weights from the original study by Mary Charlson and colleagues (1987)^1^ were used for both coding adaptations; only the International Classification of Disease (ICD) codes for each comorbidity were adapted. The US ICD‐10 coding adaptation was developed by coding experts at Optum360, IBM Watson Health, and Eli Lilly and Company, who reviewed the US adaptation of ICD codes, including but not limited to the use of ICD‐9‐CM and ICD‐10‐CM crosswalks (Centers for Medicare and Medicaid Services [CMS] general equivalency mappings [GEMs] and additional crosswalks available in Optum360°® Encoder Pro.com). Medical experts on our team considered the Deyo^2^ and Quan^14^ concepts and their categorizations (e.g., mild vs. moderate or severe) to determine which to include in each Charlson category. The full code lists are provided in Table S1. Coding trends were evaluated in a US claims data source, the IBM® MarketScan® Research Databases, during the fourth quarters of 2014 (ICD‐9) and 2015 (ICD‐10).^17^

### 2.2 Data sources

This validation was performed using 2 IBM MarketScan Research Databases—the Commercial Claims and Encounters Database and the Medicare Supplemental Database. These databases include the healthcare experience of approximately 25 million unique individuals annually, reflecting the combined healthcare service use of individuals covered in a variety of health plan types across the US. The study sample was limited to a subset of patients with data available in the IBM MarketScan Mortality File.

The Commercial Claims and Encounters Database primarily contains the claims of patients covered by employer‐sponsored health plans. The Medicare Supplemental Database includes the claims of Medicare beneficiaries with employer‐sponsored supplemental coverage. The two databases are the same in terms of their structure and available data fields. Both contain inpatient, outpatient, and pharmacy claims, including any claims paid under a coordination‐of‐benefit arrangement, and therefore provide a complete assessment of patients' healthcare resource utilization and costs covered by insurers. The IBM MarketScan Mortality File is developed through a linkage to the Social Security Administration Master Death File (SSMDF) and includes the presence and date of death. The IBM MarketScan Mortality File is complete only for those states who submit death data to the SSMDF; therefore, the study cohorts were limited to individuals residing in those states during our study period.

A request for waiver under 45 CFR 164.512(i) was obtained from the New England Institutional Review Board before the study was conducted.

### 2.3 Study populations

Adults (ages 18 years or older) with evidence of rheumatoid arthritis (inpatient or outpatient), hip or knee replacement (inpatient), lumbar spine surgery (inpatient), AMI (inpatient), stroke (inpatient), or pneumonia (inpatient or outpatient) between October 01, 2016 and April 01, 2017 were included in the study. The patient selection criteria used to identify these conditions or procedures relied on validated algorithms^21^, ^22^, ^23^, ^24^, ^25^, ^26^ and are shown in Appendix A. These six medical conditions were selected to understand the performance of the CCI in diverse populations. The index date was the date of the patient's first diagnosis (any) code for RA; first principal diagnosis code for stroke, AMI, and pneumonia; or first hospital admission date for lumbar spine surgery and hip/knee replacement during the index period. For all outcome analyses except for mortality outcomes, patients were required to have at least 12 months of continuous enrollment with medical and pharmacy benefits before the index date (i.e., pre‐index period) and following the index date (i.e., post‐index period). For mortality outcomes analyses, patients who died within 12 months after the index date were permitted and were followed until date of death. Patients with capitated claims were flagged and a gross pay amount was assigned to capitated services with a pay proxy, which used noncapitated claims and was specific to US region, year, and current procedural terminology (CPT) code (where applicable).

### 2.4 Outcome measures

Predictive performance was measured for 1‐year and postoperative mortality, in‐hospital complication, 1‐year all‐cause medical costs, and index hospitalizations costs and LOS. The CCI score and outcomes were developed using all claims during a 6‐month pre‐index period.

One‐year mortality (the proportion of patients who died within 1 year of index date) was measured using the IBM MarketScan Mortality File. All costs were assessed using fully adjudicated and paid amounts. All‐cause medical costs included inpatient, outpatient (including emergency room, office visits, and other outpatient), prescription, and all other healthcare costs during the 1‐year post‐index period. All costs were adjusted for inflation using the medical care component of the Consumer Price Index and standardized to 2018 US dollars.

For cohorts defined by operative procedures, postoperative mortality was defined as death occurring either during the index inpatient admission or within a 6‐week period following hospital discharge date.^2^ In‐hospital complications were captured using ICD‐10‐CM diagnosis and diagnosis‐related group codes for complications of treatment. Complications were identified as conditions that were not the principal causes of the inpatient admission and included serious infections and transfusions (see Appendix B for a full list of conditions and codes). Hospital LOS and costs were calculated for the index hospitalization for any patient whose index event was identified on an inpatient claim.

### 2.5 Statistical analyses

The predictive abilities of the US and Canadian adaptations were measured using methods appropriate for each type of outcome. For outcomes of 1‐year mortality, postoperative mortality, and in‐hospital complications, the area under the receiver operating characteristic curve (c‐statistic) was calculated. For outcomes of hospitalization LOS, hospitalization costs, and 1‐year all‐cause medical costs, the root mean square prediction error (RMSE) between actual and predicted values was calculated. The rationale for selecting these performance statistics was relevance and ease of interpretation for the target audience for this research (i.e., researchers using the CCI in US healthcare claims data).

Generalized linear regression models were fit to patient‐level data to calculate predicted outcomes for comparison with actual outcomes. Each model included the numeric CCI score, age, and sex as explanatory items. For binary outcomes, the model included a binomial error with logistic link; for LOS, the model included a negative binomial error with log link; and for cost outcomes, the model included a gamma error with log link.

Statistical significance of the difference in performance between the two CCI versions was determined by the method of DeLong et al (1988)^27^ paired analysis for binary outcomes. For continuous outcomes of cost and LOS, statistically significant differences were determined by resampling mean square error differences with 1000 replications, with replacement, to identify differences for which the 95% confidence interval crossed zero. *p* values <0.05 were considered significant. Reported *p*‐values were not adjusted for multiple comparisons. Statistical analyses were conducted in SAS® (version 9.4) statistical package (SAS Institute Inc., Cary, North Carolina).

## 3 RESULTS

A total of 123 626 patients met the study inclusion criteria across the six disease cohorts (Table 1). The characteristics of studied individuals are shown in Table 2. The mean age of patients was approximately 55–65 years across the cohorts. Patient sex was predominantly male in the AMI and stroke cohorts, female in the rheumatoid arthritis cohort, and evenly balanced in the hip or knee replacement, lumbar spine surgery, and pneumonia cohorts. A majority were enrolled in exclusive provider organization/preferred provider organization (EPO/PPO) plans. A substantial portion of patients resided in the South, with relatively few patients in the West, reflecting the underlying demographics of the commercial and Medicare supplemental data within the IBM MarketScan Research Databases.

**TABLE 1:** Attrition of cohorts

Variable	Rheumatoid arthritis *N* (%)	Hip or knee replacement *N* (%)	Lumbar spine surgery *N* (%)	Acute myocardial infarction *N* (%)	Stroke *N* (%)	Pneumonia N (%)
Patients in MarketScan® CCAE/MDCR with conditions of interest between October 1, 2016 and April 1, 2017 and in SSMDF	37 856 (100)	30 024 (100)	12 005 (100)	9053 (100)	7603 (100)	129 955 (100)
≥18 years of age on the index date	37 829 (99.9)	30 004 (99.9)	11 619 (96.8)	9053 (100)	7591 (99.8)	108 366 (83.4)
Continuous enrollment with medical and pharmacy benefits in the 12 months prior to the index date	29 665 (78.4)	25 811 (86.0)	9611 (80.1)	7638 (84.4)	6472 (85.1)	89 578 (68.9)
Continuous enrollment with medical and pharmacy benefits in the 12 months after the index date or deceased in SSMDF^a^	23 555 (62.2)	19 322 (64.4)	7034 (58.6)	5491 (60.7)	4244 (55.8)	63 980 (49.2)

Abbreviations: CCAE/MDCR, Commercial and Medicare supplemental databases; N, number of patients in the cohort; SSMDF, Social Security Administration Master Death File.

^a^ For all outcome analyses except for mortality outcomes, patients were required to have at least 12 months of continuous enrollment with medical and pharmacy benefits following the index date as well as prior to the index date (12‐month pre‐index period). For mortality outcomes analyses, patients who died within 12 months following the index date were permitted and were followed until date of death.

**TABLE 2:** Cohort demographics and baseline characteristics

	Rheumatoid arthritis *N* = 23 555	Hip or knee replacement *N* = 19 322	Lumbar spine surgery *N* = 7034	Acute myocardial infarction *N* = 5491	Stroke *N* = 4244	Pneumonia *N* = 63 980
Variable	Mean (SD) or *N* (%)	Mean (SD) or *N* (%)	Mean (SD) or *N* (%)	Mean (SD) or *N* (%)	Mean (SD) or *N* (%)	Mean (SD) or *N* (%)
Age	55.6 (11.5)	62.2 (9.8)	56.6 (13.3)	62.3 (12.7)	65.0 (14.4)	56.9 (17.1)
Male	5697 (24.2)	9251 (47.9)	3543 (50.4)	3980 (72.5)	2518 (59.3)	31 526 (49.3)
Geographic region						
Northeast	5961 (25.3)	4510 (23.3)	1261 (17.9)	1287 (23.4)	984 (23.2)	15 461 (24.2)
North central	4002 (17.0)	5440 (28.2)	1885 (26.8)	1500 (27.3)	1202 (28.3)	17 393 (27.2)
South	11 275 (47.9)	7350 (38.0)	3026 (43.0)	2159 (39.3)	1596 (37.6)	24 095 (37.7)
West	2268 (9.6)	1975 (10.2)	845 (12.0)	536 (9.8)	455 (10.7)	6908 (10.8)
Unknown	49 (0.2)	47 (0.2)	17 (0.2)	9 (0.2)	7 (0.2)	123 (0.2)
Insurance plan type						
Comprehensive/indemnity	1609 (6.8)	3624 (18.8)	1194 (17.0)	1217 (22.2)	1219 (28.7)	11 593 (18.1)
EPO/PPO	14 797 (62.8)	10 297 (53.3)	3804 (54.1)	2852 (51.9)	2008 (47.3)	34 358 (53.7)
POS/POS with capitation	911 (3.9)	760 (3.9)	298 (4.2)	217 (4.0)	150 (3.5)	2593 (4.1)
HMO	2235 (9.5)	1849 (9.6)	649 (9.2)	434 (7.9)	350 (8.2)	5707 (8.9)
CDHP/HDHP	3829 (16.3)	2650 (13.7)	1042 (14.8)	727 (13.2)	488 (11.5)	9308 (14.5)
Missing/unknown	174 (0.7)	142 (0.7)	47 (0.7)	44 (0.8)	29 (0.7)	421 (0.7)
Population density						
Urban	20 504 (87.0)	16 494 (85.4)	6018 (85.6)	4576 (83.3)	3704 (87.3)	55 255 (86.4)
Rural	3004 (12.8)	2786 (14.4)	1004 (14.3)	907 (16.5)	533 (12.6)	8648 (13.5)
Unknown	47 (0.2)	42 (0.2)	12 (0.2)	8 (0.1)	7 (0.2)	77 (0.1)
**Charlson comorbidities in the 6 months pre‐index**						
CCI‐US adaptation	1.40 (1.30)	0.87 (1.41)	1.15 (1.85)	1.49 (2.10)	1.71 (2.19)	1.44 (2.19)
CCI‐Canadian adaptation	1.39 (1.26)	0.83 (1.37)	1.10 (1.80)	1.42 (2.04)	1.61 (2.11)	1.38 (2.14)
Cerebrovascular disease	601 (2.6)	709 (3.7)	406 (5.8)	355 (6.5)	1087 (25.6)	4044 (6.3)
CPD	2689 (11.4)	2249 (11.6)	926 (13.2)	645 (11.7)	442 (10.4)	16 006 (25.0)
Congestive heart failure	533 (2.3)	696 (3.6)	271 (3.9)	557 (10.1)	375 (8.8)	5867 (9.2)
Dementia	74 (0.3)	159 (0.8)	57 (0.8)	92 (1.7)	188 (4.4)	2021 (3.2)
Diabetes without chronic complications	2948 (12.5)	3516 (18.2)	1395 (19.8)	1506 (27.4)	1116 (26.3)	11 565 (18.1)
Diabetes with chronic complications	841 (3.6)	966 (5.0)	489 (7.0)	629 (11.5)	540 (12.7)	4683 (7.3)
Hemiplegia/paraplegia	40 (0.2)	31 (0.2)	85 (1.2)	21 (0.4)	163 (3.8)	540 (0.8)
HIV/AIDS	9 (0.0)	26 (0.1)	37 (0.5)	24 (0.4)	8 (0.2)	262 (0.4)
Mild liver disease	746 (3.2)	503 (2.6)	297 (4.2)	158 (2.9)	115 (2.7)	2310 (3.6)
Moderate/severe liver disease	17 (0.1)	27 (0.1)	19 (0.3)	12 (0.2)	14 (0.3)	246 (0.4)
Any malignancy^a^	1017 (4.3)	1188 (6.1)	542 (7.7)	330 (6.0)	310 (7.3)	5903 (9.2)
Metastatic solid tumor	74 (0.3)	75 (0.4)	89 (1.3)	37 (0.7)	60 (1.4)	1059 (1.7)
Myocardial infarction	194 (0.8)	262 (1.4)	125 (1.8)	830 (15.1)	126 (3.0)	1684 (2.6)
Peptic ulcer disease	136 (0.6)	132 (0.7)	71 (1.0)	30 (0.5)	21 (0.5)	503 (0.8)
PVD	826 (3.5)	989 (5.1)	434 (6.2)	466 (8.5)	377 (8.9)	4769 (7.5)
Renal disease	758 (3.2)	898 (4.6)	392 (5.6)	533 (9.7)	411 (9.7)	5131 (8.0)
Rheumatic disease	18 486 (78.5)^b^	732 (3.8)	261 (3.7)	120 (2.2)	96 (2.3)	2153 (3.4)

Abbreviations: CCI, Charlson Comorbidity Index; CDHP, Consumer Directed Health Plan; CPD, chronic pulmonary disease; EPO, Exclusive Provider Organization; HDHP, High Deductible Health Plan; HIV/AIDS, human immunodeficiency virus/acquired immunodeficiency syndrome; HMO, Health Maintenance Organization; *N*, number of patients in the cohort; POS, Point of Service; PPO, Preferred Provider Organization; PVD, peripheral vascular disease; SD, standard deviation.

^a^ Any malignancy, including lymphoma and leukemia, except malignant neoplasm of skin.

^b^ Rheumatic disease is both a comorbidity in the CCI and the study cohort in this instance, which explains the high frequency.

Frequencies of pre‐index Charlson comorbidities are shown in Table 2. Our US ICD‐10 CCI adaptation produced a slightly higher index CCI score (means ranging from 0.87 to 1.71) than the Canadian adaptation (means ranging from 0.83 to 1.61; Table 2). The most commonly observed comorbidities across cohorts were chronic pulmonary disease (ranging from 10.4% to 25.0%) and diabetes without chronic complications (ranging from 12.5% to 27.4%; Table 2). Although not a comorbidity in the context of RA, rheumatologic disease codes were commonly observed in the RA cohort as could be expected.

The stroke cohort had the highest 1‐year mortality rate (10.6%), while the rheumatoid arthritis cohort had the lowest (0.2%) (Table 3). The proportion of patients with postoperative mortality was 1.1% and 0.2% among the lumbar spine surgery and hip or knee replacement cohorts, respectively. The pneumonia cohort had the highest proportion of patients with in‐hospital complications (75.1%), while the hip or knee replacement cohort had the lowest proportion (8.5%). Mean 1‐year medical costs ranged from $38 116 in the pneumonia cohort to $113 505 in the lumbar spine surgery cohort. Among patients with an inpatient admission, mean hospitalization costs ranged from $25 961 in the pneumonia cohort to $70 526 in the lumbar spine surgery cohort and the mean LOS for the index hospitalization ranged from 2.38 days for the hip or knee replacement cohort to 5.05 days for the pneumonia cohort.

**TABLE 3:** Descriptive summary of outcomes

Cohort	*N* ^a^	1‐year mortality^b^ (%)	Hosp mortality^c^ (%)	*N* ^d^	Hosp complication^e^ (%)	*N* ^f^	1‐year cost^g^ US ($)	*N* ^h^	Hosp LOS^i^ days	Hosp cost^j^ US ($)
Rheumatoid arthritis	23 555	0.2	N/A	2491	41.0	23 510	45 055	146	3.6	35 873
Hip or knee replacement	19 322	0.5	0.2	19 243	8.5	19 243	65 513	19 243	2.4	39 060
Lumbar spine surgery	7034	2.1	1.1	6895	21.4	6895	113 505	6895	4.1	70 526
Acute myocardial infarction	5491	6.4	N/A	5149	24.2	5149	95 399	5149	3.8	47 753
Stroke	4244	10.6	N/A	3809	25.1	3809	92 170	3809	4.9	37 777
Pneumonia	63 980	3.1	N/A	16 271	75.1	62 038	38 116	5828	5.1	25 961

Abbreviations: Hosp, hospital; LOS, length of stay; *N*, number of patients in the cohort; N/A, not applicable.

^a^ The 1‐year mortality analyses include patients with evidence of death during the 12‐month follow‐up period (Social Security Administration death master file) or at least 12 months of follow‐up time.

^b^ One‐year mortality was defined as dying within 12‐month follow‐up.

^c^ In‐hospital mortality was defined as dying during the index inpatient admission or within a 6‐week period following hospital discharge.

^d^ Hospital complications analyses include patients with 12‐month follow‐up. Patients in pneumonia and rheumatoid arthritis cohorts without an inpatient admission during the 12‐month follow‐up period were excluded from this analysis.

^e^ Hospital complications were defined as having an ICD‐10‐CM diagnosis for infection, ICD‐10 procedure for blood transfusion, or diagnosis‐related group for complications of treatment during the index hospitalization (see Appendix B for full list of concepts and codes).

^f^ One‐year medical cost analyses include patients with 12 months of follow‐up time.

^g^ Cost is represented as means and were adjusted for inflation using the medical care component of the Consumer Price Index and standardized to 2018 US dollars.

^h^ Hospital LOS and cost analyses includes patients with 12 months of follow‐up time and hospitalization on the index date and includes patients who did not have a hospitalization.

^i^ Hospital LOS is represented as mean days of the index hospitalization.

^j^ Hospital costs are represented as means for the index hospitalization and adjusted for inflation using the medical care component of the Consumer Price Index and standardized to 2018 US dollars.

**FIGURE 1:**
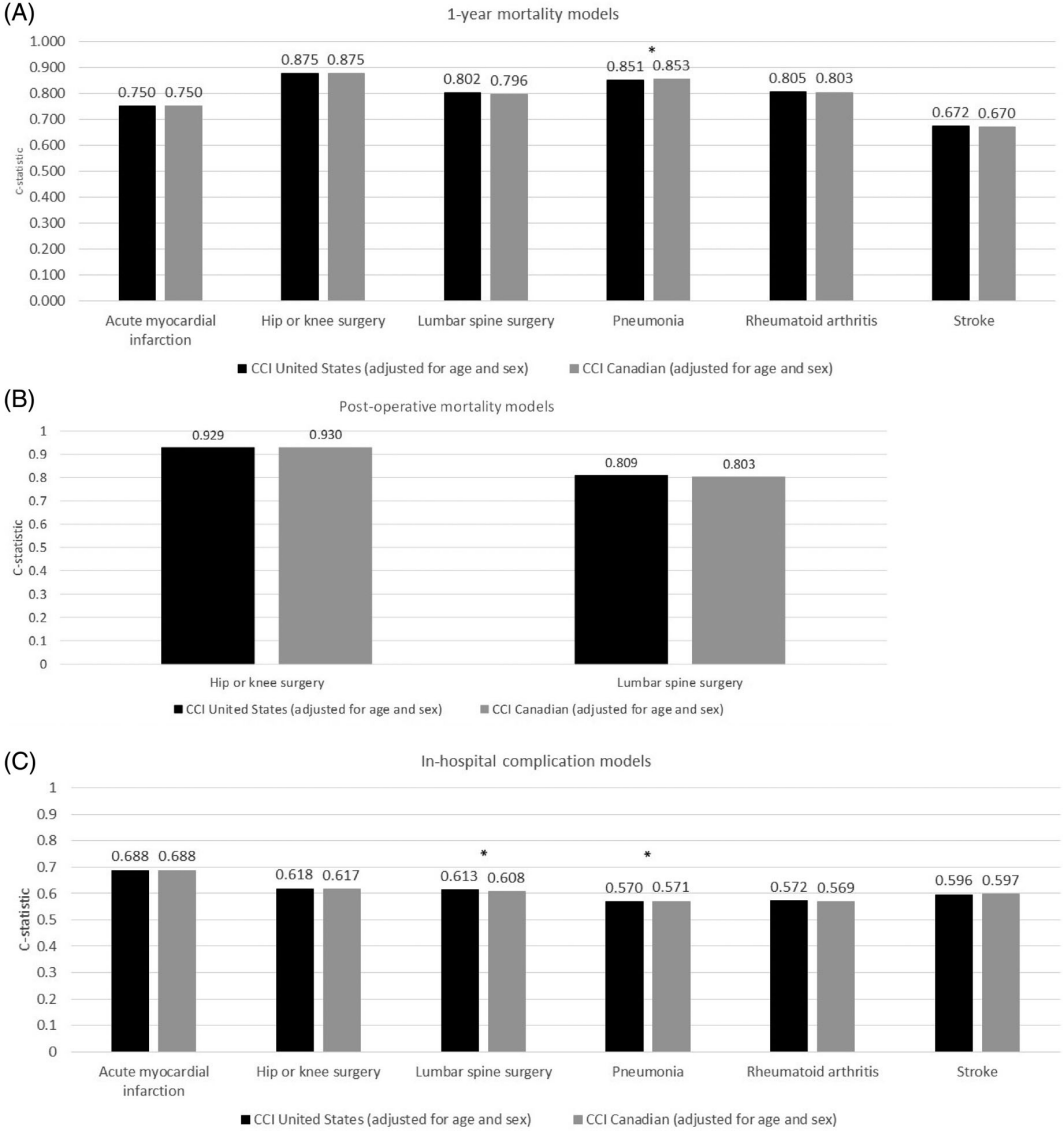
Performance (c‐statistics) of models predicting 1‐year mortality (A), in‐hospital mortality (B), and in‐hospital complications (C). Patients included in the 1‐year mortality model are those with evidence of death during the 12‐month follow‐up period (Social Security Administration death master file) or at least 12 months of follow‐up time. One‐year mortality was defined as death within the 12‐month follow‐up period. (A). Patients included in the in‐hospital mortality model are those with evidence of death during the 12‐month follow‐up period (Social Security Administration death master file) or at least 12 months of follow‐up time. Postoperative mortality was defined as death during the index inpatient admission or within a 6‐week period following hospital discharge (B). In‐hospital complication models include patients with 12 months of follow‐up time. Patients in pneumonia and rheumatoid arthritis cohorts without an inpatient admission during the 12‐month follow‐up period were excluded from the in‐hospital complication model. In‐hospital complication was defined as having an ICD‐10‐CM diagnosis for infection, ICD‐10 procedure for blood transfusion, or diagnosis‐related group for complications of treatment (see Appendix B for full list of concepts and codes) (C). All models include as explanatory items the numeric CCI score, numeric age, and indicator of female sex. The CCI score was calculated based on both inpatient and outpatient claims during the 6‐month period prior to Index. * denotes *p* values <0.05 for the comparison of the US and Canadian adaptations. CCI, Charlson Comorbidity Index

C‐statistics across cohorts for 1‐year mortality models ranged from 0.67 to 0.87 (Figure 1(A)) and for postoperative mortality models ranged from 0.80 to 0.93 (Figure 1(B)). Mortality c‐statistics were similar between the US and Canadian coding adaptations with relative percentage differences in the range of −0.17% to 0.79% (Table 4). C‐statistics for in‐hospital complications models ranged from 0.57 to 0.69 (Figure 1(C)), with relative percentage differences ranging from −0.32% to 0.76% (Table 4).

**TABLE 4:** Percentage difference^a^ in predictive ability of US versus Canadian CCI adaptations

Condition	1‐year mortality	Hospital complication	Postoperative mortality	1‐year total cost	Hospital LOS	Hospital cost
Acute myocardial infarction	0.10%	0.10%	N/A	−0.33%	0.02%	−0.01%
Hip or knee replacement	−0.02%	0.19%	−0.07%	−0.32%	−0.05%	−0.02%
Lumbar spine surgery	0.79%	0.76%	0.69%	−0.14%	0.45%	0.02%
Pneumonia	−0.17%	−0.24%	N/A	−5.61%^b^	−0.04%	0.01%
Rheumatoid arthritis	0.30%	0.53%	N/A	−0.24%	−0.26%	0.22%
Stroke	0.26%	−0.32%	N/A	0.20%	0.01%	−0.02%

Abbreviations: LOS, length of stay; N/A, not applicable.

^a^ Percentage difference in c‐statistics for 1‐year mortality, in‐hospital mortality, and hospital complications during the index hospitalization and in root mean square prediction error (RMSE) for 1‐year medical costs, hospital LOS for the index hospitalization, and hospital costs for the index hospitalization. Calculated as the US adaptation minus the Canadian adaptation, divided by the Canadian adaptation and expressed as a percentage.

^b^ 95% confidence interval for difference in predictive ability for 1‐year total cost for pneumonia = (−11.97%, 0.75%).

**FIGURE 2:**
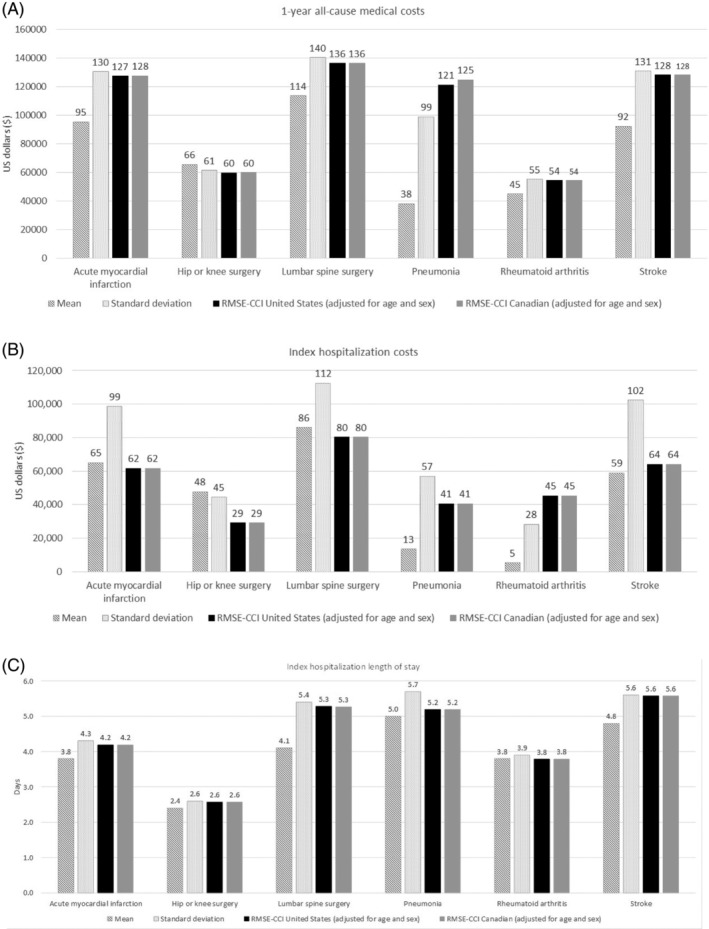
Performance (root mean square errors) of models predicting 1‐year all‐cause medical costs (A), index hospitalization costs (B), and index hospitalization length of stay (C). Patients included in the 1‐year all‐cause cost models are those with 12 months of follow‐up time. Costs were adjusted for inflation using the medical care component of the Consumer Price Index and standardized to 2018 US dollars (A). Patients included in the index hospitalization costs modes are those with 12 months of follow‐up time and hospitalization on the index date. Costs were adjusted for inflation using the medical care component of the Consumer Price Index and standardized to 2018 US dollars (B). Patients included in the index hospitalization length of stay models are those with 12 months of follow‐up time and hospitalization on the index date (C). All models include as explanatory items the numeric CCI score, numeric age, and indicator of female sex. The CCI score was calculated based on both inpatient and outpatient claims during the 6‐month period prior to Index. RMSE is the root mean square error (MSE) of total cost in $ thousands or length of stay in days. CCI, Charlson Comorbidity Index; RMSE, root mean square error

For both coding adaptations, the RMSEs for 1‐year medical costs, index hospitalization costs, and LOS were relatively large, similar to the mean and standard deviations (Figure 2(A–C)). The relative percentage RMSE differences for 1‐year medical costs ranged from −5.61% in the pneumonia cohort to 0.20% in the stroke cohort (Table 4). The relative percentage differences for hospitalization costs and LOS for the index hospitalization ranged from −0.26% to 0.45% (Table 4). Statistically significant differences were observed between coding adaptations for the pneumonia cohort for 1‐year mortality and in‐hospital complications and for the lumbar spine surgery cohort for in‐hospital complications.

## 4 DISCUSSION

The US and Canadian ICD‐10 coding adaptations of the CCI, including age and sex effects, performed similarly in this study of diverse patient groups and outcomes in US healthcare commercial claims. Both increases and decreases of very small magnitude in predictive ability were observed when comparing the performance metrics of our US‐based adaptation versus the Canadian adaptation. A few of the comparisons resulted in *p* values <0.05, but this could be expected in the context of large sample sizes and many statistical tests performed. Greater variation in c‐statistics and RMSE was observed across cohorts than between the US and Canadian coding adaptations, evidence that the predictive ability of the algorithms had more to do with the underlying medical conditions than the ICD‐10 code sets used to create the CCI.

For the binary outcome measures, c‐statistics of 0.8 and above are generally considered well discriminating, while 0.6 and below are considered poorly discriminating.^28^ The c‐statistic exceeded 0.8 for the mortality outcomes (1‐year and postoperative mortality) for many cohorts. The exceptions were 1‐year mortality c‐statistics for the stroke and AMI cohorts; the smaller (<0.8) c‐statistics observed could be expected for medical conditions in the emergency care setting where other factors not assessed in claims (e.g., the severity and distribution of vascular disease and health delivery factors like how quickly the patient receives care and where it is received) are likely to have a greater impact on mortality outcomes. Smaller (<0.8) c‐statistics were observed for in‐hospital complications, indicating worse predictive ability for in‐hospital complications than for mortality; however, the predictive ability of each model was consistently similar between the US and Canadian adaptations. The definition of in‐hospital complications was different in this study than that used in a previous ICD‐9‐CM validation. Deyo and colleagues (1992)^2^ tailored the definition of complications to a single study cohort of lumbar spine surgery patients while this study defined complications more broadly to apply across multiple and diverse cohorts, which may explain the poorer predictive ability observed for the in‐hospital complication models in our study.

For the continuous outcome measures, RMSE is a frequently used and widely recognized measure, but there is no single threshold to define good predictive ability of these models (i.e., how well predicted costs correspond with observed costs). The RMSEs for 1‐year and index hospitalization medical costs and LOS were relatively large, representing a large proportion of the means, and similar in magnitude to the standard deviation; we interpreted this as poor predictive ability of the models. However, again, similar results were observed for both the US and Canadian adaptations.

Because of different codes or categorization of codes in some cases, our US CCI coding adaptation produced slightly higher index scores than the Canadian coding adaptation (Table 2). While there were only two codes in the publication by Quan et al^14^ that did not appear in our coding adaptation, there were over 400 codes in our coding adaptation that either do not appear in the Quan et al publication or are included in a different category (e.g., differences in interpretation between “diabetes with chronic complications” and “diabetes without chronic complications”). Some differences in coding were due to different interpretations of the code range implied in Quan's publication, Table 1 (e.g., E10.6 is included but not E10.6*, which we interpreted to mean a single E10.6 code only and not including the full range of codes under E10.6). To promote clarity in the interpretation of code lists, we provide a machine‐readable list of our individual ICD‐10 codes along with the codes from Quan's publication used in this study (https://doi.org/10.5281/zenodo.3604394) as well as the full code list in Table S1. However, some differences in coding are due to different ICD‐10 coding adaptations between Canada and the US. Some codes in the US ICD‐10 system represent different concepts or do not exist in the Canadian ICD‐10 system. For example, US billable ICD‐10 codes that specify “with coma” or “without coma” (e.g., E13.10, E13.11, E13.649, E13.641, E13.00, E13.01, K72.90, K72.91, K72.10, K72.11, K70.40, K70.41) were not present in the Canadian ICD‐10 adaptation. Because the number of relevant codes in the US exceeds the number of relevant codes in the Canadian ICD‐10 system for some comorbidities, there are more opportunities to identify the comorbidity in US data and increase the score.

We do not know whether using the Canadian code set results in erroneous assignment of individual comorbidities since we did not evaluate construct validity of the CCI codes (e.g., verification of individual comorbidities through medical chart review). However, there is a potential for worse construct validity when using a coding adaptation to identify concepts in a data source from a different country, given differences in coding adaptations and real world reimbursement environments.^16^

An important question and limitation of the current study concerns future updates of Charlson comorbidity codes over time as newer ICD‐10 concepts and codes are introduced in the US. For example, the ICD‐9‐CM Charlson adaptation by Deyo and colleagues^2^ does not include some codes for dementia (i.e., 294.10, 294.11, 294.20, 294.21, 331.0, 331.11) or mild liver disease (i.e., 070.22, 070.23, 070.32, 070.33, 070.44, 070.54, 070.6, 070.9, 570, 571.0, 571.1, 571.3, 571.8). Many of these codes became effective in US healthcare claims after the time of Deyo's study. Omitting these codes has been shown to appreciably decrease the observed rate of comorbidities in US healthcare claims.^17^ Our US adaptation represents a point in time early in the adoption and evolution of ICD‐10, but future changes in ICD will be important for researchers to review and consider when deciding how to use this coding adaptation.

Our intent was not to evaluate whether the original Charlson concepts or weights can be improved upon; others have considered these issues.^29^, ^30^, ^31^, ^32^, ^33^ Our aim was to evaluate the predictive validity of an ICD‐10 coding adaptation for Charlson comorbidities in US healthcare data and compare it with a standard that many researchers in the US were likely using in the absence of a previously validated US coding adaptation. In general, both the US and Canadian adaptations performed well and similarly in this study.

## 5 CONCLUSION

This US‐based coding adaptation and a previously published Canadian adaptation^14^ performed similarly well for mortality outcomes and more poorly for in‐hospital complications, costs, and LOS. While both ICD‐10 CCI coding adaptations resulted in similar c‐statistics and RMSEs (predictive ability) in these US healthcare data, using the Canadian adaptation with US claims data could result in erroneous assignment of individual comorbidities (i.e., worse construct validity) given different ICD‐10 coding adaptations and real world reimbursement environments in Canada and the US. Based on good research practice guidelines, we recommend using adaptations specific to the country of origin of the data.^16^

## ETHICS STATEMENT

This manuscript is the original work of all the authors. The data were presented in part (1‐year mortality outcome results) poster presentation at the 25th annual meeting of the International Society for Pharmacoeconomics and Outcomes Research (ISPOR) in May 2020 and published as an abstract in the meeting supplement (Beyrer J, Manjelievskaia J, Bonafede M, et al PMU40 validation of an ICD‐10 coding adaption for the Charlson Comorbidity Index in United States healthcare administrative data. *Value Health*. 2020;23 (Suppl 1):S240. https://doi.org/10.1016/j.jval.2020.04.814).

## CONFLICT OF INTEREST

Julie Beyrer, Sandra Nolot, Diane Haldane, and Joseph Johnston are employees and shareholders of Eli Lilly and Company. Machaon Bonafede, Janna Manjelievskaia, and Gregory Lenhart are employees of IBM Watson Health.

## Supporting information


**Table S1.** ICD‐10 codes used to define each Charlson comorbidity.

## Appendix

Specific medical codes used to identify the cohorts are available in machine readable format on zenodo.org (https://doi.org/10.5281/zenodo.3968742).

### Rheumatoid arthritis (RA)

Patients will be identified as having RA based on an adaptation of published claims‐based algorithms with high positive predictive values (PPVs).^23^ Patients will be required to have an ICD‐10‐CM diagnosis code for RA, an encounter with a rheumatologist, and evidence of disease‐modifying antirheumatic drug (DMARD) use during the study period in either an inpatient or outpatient setting. In the outpatient setting, to determine whether to use all outpatient claims or restrict to non‐diagnostic claims on professional medical encounters, separate sample counts will be calculated and compared. Depending on sample size, patients may not be required to have evidence of a rheumatologist encounter to be included in the RA cohort.

### Hip or knee replacement

Patients will be identified as having undergone hip or knee replacement based on a validated algorithm using administrative data (ICD‐9‐CM) with a PPV of 95% for knee replacements and 98% for hip replacements^22^ and adapted to ICD‐10‐CM. Patients will be required to have an inpatient claim with an ICD‐10‐CM diagnosis or procedure (Current Procedural Terminology (CPT), ICD‐10‐PCS) code for knee or hip replacement during the study period.

### Lumbar spine surgery

Patients will be identified as having lumbar spine surgery based on a validated claims‐based algorithm (using ICD‐9‐CM)^25^ and adapted to ICD‐10‐CM. Patients will be required to have an inpatient procedure (CPT, ICD‐10‐PCS) code indicating lumbar fusion, with or without decompression, for back pain, herniated disc, stenosis, spondylolisthesis or scoliosis during the study period. The specificity of this algorithm ranges from 85.6% to 97.3%, depending on the surgery indication.^25^

### Acute myocardial infarction

Patients will be identified as having AMI based on a validated algorithm using administrative data with a PPV of 82.2%.^24^ Patients will be required to have an inpatient claim with an ICD‐10‐CM diagnosis for AMI (I21‐I22) in the primary diagnosis position during the study period.

### Stroke

Patients will be identified as having a stroke based on a systematic review analyzing the validity of stroke codes in administrative databases.^26^ Patients will be required to have an inpatient claim with a diagnosis of acute stroke (ICD‐10‐CM I60, I61, I63) during the study period, as these codes are highly predictive of true cases of acute stroke.^26^

### Pneumonia

To the best of our knowledge, there is a lack of validated U.S. claims‐based algorithms to identify pneumonia with a high PPV. Aronsky et al (2005)^21^ examined the accuracy of administrative data for identifying patients with pneumonia using ICD‐9‐CM diagnosis and diagnosis‐related group (DRG) codes and found that the sensitivity ranged from 47.8% to 66.2% and PPV ranged from 72.6% to 80.8%, with DRG codes being significantly less precise in identifying pneumonia patients. For this study, we propose identifying pneumonia patients based on the Centers for Medicare and Medicaid Services' (CMS) method of using ICD‐10‐CM diagnosis codes for pneumonia in the primary position in an inpatient or outpatient setting.^30^

## Appendix

Codes used to define in‐hospital complications are available in machine readable format on zenodo.org (https://doi.org/10.5281/zenodo.3968784).

## Appendix

Codes used to define each Charlson Comorbidity are available in machine readable format on zenodo.org (https://doi.org/10.5281/zenodo.3604394) or in the tabular format shown in Table S1. SAS programs for implementing this CCI are available here: https://github.com/beyrerj/US_ICD10_Charlsonindex
