# Supplementary material for: Validation of an International Classification of Disease, 10th revision coding adaptation for the Charlson Comorbidity Index in United States healthcare claims data
Source: Pharmacoepidemiol Drug Saf. 2021 Mar 4;30(5):582–93. doi: 10.1002/pds.5204 (PMC8252530; doi:10.1002/pds.5204)
Supplement: Supplementary file 1 — Table S1. ICD‐10 codes used to define each Charlson comorbidity. [file PDS-30-582-s001.docx]

**Appendix/Supplemental Table 1: ICD-10 codes used to define each Charlson comorbidity**

| **Charlson comorbidity** | **United States CCI adaptation** | **Canadian CCI adaptation^a^** |
| --- | --- | --- |
| Myocardial infarction | **ICD-10-CM:**  I2101  I2102  I2109  I2111  I2119  I2121  I2129  I213  I214  I219  I21A1  I21A9  I220  I221  I222  I228  I229  I252 | **ICD-10-CM:**  I2101  I2102  I2109  I2111  I2119  I2121  I2129  I213  I214  I219  I21A1  I21A9  I220  I221  I222  I228  I229  I252 |
| Congestive heart failure | **ICD-10-CM:**  I099  I110  I130  I132  I255  I420  I425  I426  I427  I428  I429  I43  I501  I5020  I5021  I5022  I5023  I5030  I5031  I5032  I5033  I5040  I5041  I5042  I5043  I50810  I50811  I50812  I50813  I50814  I5082  I5083  I5084  I5089  I509  P290 | **ICD-10-CM:**  I099  I110  I130  I132  I255  I420  I425  I426  I427  I428  I429  I43  I501  I5020  I5021  I5022  I5023  I5030  I5031  I5032  I5033  I5040  I5041  I5042  I5043  I50810  I50811  I50812  I50813  I50814  I5082  I5083  I5084  I5089  I509  P290 |
| Peripheral vascular disease | **ICD-10-CM:**  A5201  I700  I701  I70201  I70202  I70203  I70208  I70209  I70211  I70212  I70213  I70218  I70219  I70221  I70222  I70223  I70228  I70229  I70231  I70232  I70233  I70234  I70235  I70238  I70239  I70241  I70242  I70243  I70244  I70245  I70248  I70249  I7025  I70261  I70262  I70263  I70268  I70269  I70291  I70292  I70293  I70298  I70299  I70301  I70302  I70303  I70308  I70309  I70311  I70312  I70313  I70318  I70319  I70321  I70322  I70323  I70328  I70329  I70331  I70332  I70333  I70334  I70335  I70338  I70339  I70341  I70342  I70343  I70344  I70345  I70348  I70349  I7035  I70361  I70362  I70363  I70368  I70369  I70391  I70392  I70393  I70398  I70399  I70401  I70402  I70403  I70408  I70409  I70411  I70412  I70413  I70418  I70419  I70421  I70422  I70423  I70428  I70429  I70431  I70432  I70433  I70434  I70435  I70438  I70439  I70441  I70442  I70443  I70444  I70445  I70448  I70449  I7045  I70461  I70462  I70463  I70468  I70469  I70491  I70492  I70493  I70498  I70499  I70501  I70502  I70503  I70508  I70509  I70511  I70512  I70513  I70518  I70519  I70521  I70522  I70523  I70528  I70529  I70531  I70532  I70533  I70534  I70535  I70538  I70539  I70541  I70542  I70543  I70544  I70545  I70548  I70549  I7055  I70561  I70562  I70563  I70568  I70569  I70591  I70592  I70593  I70598  I70599  I70601  I70602  I70603  I70608  I70609  I70611  I70612  I70613  I70618  I70619  I70621  I70622  I70623  I70628  I70629  I70631  I70632  I70633  I70634  I70635  I70638  I70639  I70641  I70642  I70643  I70644  I70645  I70648  I70649  I7065  I70661  I70662  I70663  I70668  I70669  I70691  I70692  I70693  I70698  I70699  I70701  I70702  I70703  I70708  I70709  I70711  I70712  I70713  I70718  I70719  I70721  I70722  I70723  I70728  I70729  I70731  I70732  I70733  I70734  I70735  I70738  I70739  I70741  I70742  I70743  I70744  I70745  I70748  I70749  I7075  I70761  I70762  I70763  I70768  I70769  I70791  I70792  I70793  I70798  I70799  I708  I7090  I7091  I7092  I7100  I7101  I7102  I7103  I711  I712  I713  I714  I715  I716  I718  I719  I731  I7389  I739  I75011  I75012  I75013  I75019  I75021  I75022  I75023  I75029  I7581  I7589  I771  I7770  I7771  I7772  I7773  I7774  I7775  I7776  I7777  I7779  I790  K551  K558  K559  Z95820  Z95828  **ICD-10-PCS:**  04RK07Z  04RK0JZ  04RK0KZ  04RK47Z  04RK4JZ  04RK4KZ  04RL07Z  04RL0JZ  04RL0KZ  04RL47Z  04RL4JZ  04RL4KZ  04RM07Z  04RM0JZ  04RM0KZ  04RM47Z  04RM4JZ  04RM4KZ  04RN07Z  04RN0JZ  04RN0KZ  04RN47Z  04RN4JZ  04RN4KZ  04RP07Z  04RP0JZ  04RP0KZ  04RP47Z  04RP4JZ  04RP4KZ  04RQ07Z  04RQ0JZ  04RQ0KZ  04RQ47Z  04RQ4JZ  04RQ4KZ  04RR07Z  04RR0JZ  04RR0KZ  04RR47Z  04RR4JZ  04RR4KZ  04RS07Z  04RS0JZ  04RS0KZ  04RS47Z  04RS4JZ  04RS4KZ  04RT07Z  04RT0JZ  04RT0KZ  04RT47Z  04RT4JZ  04RT4KZ  04RU07Z  04RU0JZ  04RU0KZ  04RU47Z  04RU4JZ  04RU4KZ  04RV07Z  04RV0JZ  04RV0KZ  04RV47Z  04RV4JZ  04RV4KZ  04RW07Z  04RW0JZ  04RW0KZ  04RW47Z  04RW4JZ  04RW4KZ  04RY07Z  04RY0JZ  04RY0KZ  04RY47Z  04RY4JZ  04RY4KZ  04R007Z  04R00JZ  04R00KZ  04R047Z  04R04JZ  04R04KZ  04R107Z  04R10JZ  04R10KZ  04R147Z  04R14JZ  04R14KZ  04R207Z  04R20JZ  04R20KZ  04R247Z  04R24JZ  04R24KZ  04R307Z  04R30JZ  04R30KZ  04R347Z  04R34JZ  04R34KZ  04R407Z  04R40JZ  04R40KZ  04R447Z  04R44JZ  04R44KZ  04R507Z  04R50JZ  04R50KZ  04R547Z  04R54JZ  04R54KZ  04R607Z  04R60JZ  04R60KZ  04R647Z  04R64JZ  04R64KZ  04R707Z  04R70JZ  04R70KZ  04R747Z  04R74JZ  04R74KZ  04R807Z  04R80JZ  04R80KZ  04R847Z  04R84JZ  04R84KZ  04R907Z  04R90JZ  04R90KZ  04R947Z  04R94JZ  04R94KZ  04RA07Z  04RA0JZ  04RA0KZ  04RA47Z  04RA4JZ  04RA4KZ  04RB07Z  04RB0JZ  04RB0KZ  04RB47Z  04RB4JZ  04RB4KZ  04RC07Z  04RC0JZ  04RC0KZ  04RC47Z  04RC4JZ  04RC4KZ  04RD07Z  04RD0JZ  04RD0KZ  04RD47Z  04RD4JZ  04RD4KZ  04RE07Z  04RE0JZ  04RE0KZ  04RE47Z  04RE4JZ  04RE4KZ  04RF07Z  04RF0JZ  04RF0KZ  04RF47Z  04RF4JZ  04RF4KZ  04RH07Z  04RH0JZ  04RH0KZ  04RH47Z  04RH4JZ  04RH4KZ  04RJ07Z  04RJ0JZ  04RJ0KZ  04RJ47Z  04RJ4JZ  04RJ4KZ | **ICD-10-CM:**  I700  I701  I70201  I70202  I70203  I70208  I70209  I70211  I70212  I70213  I70218  I70219  I70221  I70222  I70223  I70228  I70229  I70231  I70232  I70233  I70234  I70235  I70238  I70239  I70241  I70242  I70243  I70244  I70245  I70248  I70249  I7025  I70261  I70262  I70263  I70268  I70269  I70291  I70292  I70293  I70298  I70299  I70301  I70302  I70303  I70308  I70309  I70311  I70312  I70313  I70318  I70319  I70321  I70322  I70323  I70328  I70329  I70331  I70332  I70333  I70334  I70335  I70338  I70339  I70341  I70342  I70343  I70344  I70345  I70348  I70349  I7035  I70361  I70362  I70363  I70368  I70369  I70391  I70392  I70393  I70398  I70399  I70401  I70402  I70403  I70408  I70409  I70411  I70412  I70413  I70418  I70419  I70421  I70422  I70423  I70428  I70429  I70431  I70432  I70433  I70434  I70435  I70438  I70439  I70441  I70442  I70443  I70444  I70445  I70448  I70449  I7045  I70461  I70462  I70463  I70468  I70469  I70491  I70492  I70493  I70498  I70499  I70501  I70502  I70503  I70508  I70509  I70511  I70512  I70513  I70518  I70519  I70521  I70522  I70523  I70528  I70529  I70531  I70532  I70533  I70534  I70535  I70538  I70539  I70541  I70542  I70543  I70544  I70545  I70548  I70549  I7055  I70561  I70562  I70563  I70568  I70569  I70591  I70592  I70593  I70598  I70599  I70601  I70602  I70603  I70608  I70609  I70611  I70612  I70613  I70618  I70619  I70621  I70622  I70623  I70628  I70629  I70631  I70632  I70633  I70634  I70635  I70638  I70639  I70641  I70642  I70643  I70644  I70645  I70648  I70649  I7065  I70661  I70662  I70663  I70668  I70669  I70691  I70692  I70693  I70698  I70699  I70701  I70702  I70703  I70708  I70709  I70711  I70712  I70713  I70718  I70719  I70721  I70722  I70723  I70728  I70729  I70731  I70732  I70733  I70734  I70735  I70738  I70739  I70741  I70742  I70743  I70744  I70745  I70748  I70749  I7075  I70761  I70762  I70763  I70768  I70769  I70791  I70792  I70793  I70798  I70799  I708  I7090  I7091  I7092  I7100  I7101  I7102  I7103  I711  I712  I713  I714  I715  I716  I718  I719  I731  I739  I771  I790  K551  K558  K559  Z959 |
| Cerebrovascular disease | **ICD-10-CM:**  G450  G451  G452  G453  G454  G458  G459  G460  G461  G462  G463  G464  G465  G466  G467  G468  H3400  H3401  H3402  H3403  H3410  H3411  H3412  H3413  H34211  H34212  H34213  H34219  H34231  H34232  H34233  H34239  H348110  H348111  H348112  H348120  H348121  H348122  H348130  H348131  H348132  H348190  H348191  H348192  H34821  H34822  H34823  H34829  H348310  H348311  H348312  H348320  H348321  H348322  H348330  H348331  H348332  H348390  H348391  H348392  H349  I6000  I6001  I6002  I6010  I6011  I6012  I602  I6030  I6031  I6032  I604  I6050  I6051  I6052  I606  I607  I608  I609  I610  I611  I612  I613  I614  I615  I616  I618  I619  I6200  I6201  I6202  I6203  I621  I629  I6300  I63011  I63012  I63013  I63019  I6302  I63031  I63032  I63033  I63039  I6309  I6310  I63111  I63112  I63113  I63119  I6312  I63131  I63132  I63133  I63139  I6319  I6320  I63211  I63212  I63213  I63219  I6322  I63231  I63232  I63233  I63239  I6329  I6330  I63311  I63312  I63313  I63319  I63321  I63322  I63323  I63329  I63331  I63332  I63333  I63339  I63341  I63342  I63343  I63349  I6339  I6340  I63411  I63412  I63413  I63419  I63421  I63422  I63423  I63429  I63431  I63432  I63433  I63439  I63441  I63442  I63443  I63449  I6349  I6350  I63511  I63512  I63513  I63519  I63521  I63522  I63523  I63529  I63531  I63532  I63533  I63539  I63541  I63542  I63543  I63549  I6359  I636  I638  I639  I6501  I6502  I6503  I6509  I651  I6521  I6522  I6523  I6529  I658  I659  I6601  I6602  I6603  I6609  I6611  I6612  I6613  I6619  I6621  I6622  I6623  I6629  I663  I668  I669  I670  I671  I672  I673  I674  I675  I676  I677  I6781  I6782  I6783  I67841  I67848  I6789  I679  I680  I682  I688  I6900  I69010  I69011  I69012  I69013  I69014  I69015  I69018  I69019  I69020  I69021  I69022  I69023  I69028  I69031  I69032  I69033  I69034  I69039  I69041  I69042  I69043  I69044  I69049  I69051  I69052  I69053  I69054  I69059  I69061  I69062  I69063  I69064  I69065  I69069  I69090  I69091  I69092  I69093  I69098  I6910  I69110  I69111  I69112  I69113  I69114  I69115  I69118  I69119  I69120  I69121  I69122  I69123  I69128  I69131  I69132  I69133  I69134  I69139  I69141  I69142  I69143  I69144  I69149  I69151  I69152  I69153  I69154  I69159  I69161  I69162  I69163  I69164  I69165  I69169  I69190  I69191  I69192  I69193  I69198  I6920  I69210  I69211  I69212  I69213  I69214  I69215  I69218  I69219  I69220  I69221  I69222  I69223  I69228  I69231  I69232  I69233  I69234  I69239  I69241  I69242  I69243  I69244  I69249  I69251  I69252  I69253  I69254  I69259  I69261  I69262  I69263  I69264  I69265  I69269  I69290  I69291  I69292  I69293  I69298  I6930  I69310  I69311  I69312  I69313  I69314  I69315  I69318  I69319  I69320  I69321  I69322  I69323  I69328  I69331  I69332  I69333  I69334  I69339  I69341  I69342  I69343  I69344  I69349  I69351  I69352  I69353  I69354  I69359  I69361  I69362  I69363  I69364  I69365  I69369  I69390  I69391  I69392  I69393  I69398  I6980  I69810  I69811  I69812  I69813  I69814  I69815  I69818  I69819  I69820  I69821  I69822  I69823  I69828  I69831  I69832  I69833  I69834  I69839  I69841  I69842  I69843  I69844  I69849  I69851  I69852  I69853  I69854  I69859  I69861  I69862  I69863  I69864  I69865  I69869  I69890  I69891  I69892  I69893  I69898  I6990  I69910  I69911  I69912  I69913  I69914  I69915  I69918  I69919  I69920  I69921  I69922  I69923  I69928  I69931  I69932  I69933  I69934  I69939  I69941  I69942  I69943  I69944  I69949  I69951  I69952  I69953  I69954  I69959  I69961  I69962  I69963  I69964  I69965  I69969  I69990  I69991  I69992  I69993  I69998 | **ICD-10-CM:**  G450  G451  G452  G453  G454  G458  G459  G460  G461  G462  G463  G464  G465  G466  G467  G468  I6000  I6001  I6002  I6010  I6011  I6012  I602  I6030  I6031  I6032  I604  I6050  I6051  I6052  I606  I607  I608  I609  I610  I611  I612  I613  I614  I615  I616  I618  I619  I6200  I6201  I6202  I6203  I621  I629  I6300  I63011  I63012  I63013  I63019  I6302  I63031  I63032  I63033  I63039  I6309  I6310  I63111  I63112  I63113  I63119  I6312  I63131  I63132  I63133  I63139  I6319  I6320  I63211  I63212  I63213  I63219  I6322  I63231  I63232  I63233  I63239  I6329  I6330  I63311  I63312  I63313  I63319  I63321  I63322  I63323  I63329  I63331  I63332  I63333  I63339  I63341  I63342  I63343  I63349  I6339  I6340  I63411  I63412  I63413  I63419  I63421  I63422  I63423  I63429  I63431  I63432  I63433  I63439  I63441  I63442  I63443  I63449  I6349  I6350  I63511  I63512  I63513  I63519  I63521  I63522  I63523  I63529  I63531  I63532  I63533  I63539  I63541  I63542  I63543  I63549  I6359  I636  I638  I639  I6501  I6502  I6503  I6509  I651  I6521  I6522  I6523  I6529  I658  I659  I6601  I6602  I6603  I6609  I6611  I6612  I6613  I6619  I6621  I6622  I6623  I6629  I663  I668  I669  I670  I671  I672  I673  I674  I675  I676  I677  I6781  I6782  I6783  I67841  I67848  I6789  I679  I680  I682  I688  I6900  I69010  I69011  I69012  I69013  I69014  I69015  I69018  I69019  I69020  I69021  I69022  I69023  I69028  I69031  I69032  I69033  I69034  I69039  I69041  I69042  I69043  I69044  I69049  I69051  I69052  I69053  I69054  I69059  I69061  I69062  I69063  I69064  I69065  I69069  I69090  I69091  I69092  I69093  I69098  I6910  I69110  I69111  I69112  I69113  I69114  I69115  I69118  I69119  I69120  I69121  I69122  I69123  I69128  I69131  I69132  I69133  I69134  I69139  I69141  I69142  I69143  I69144  I69149  I69151  I69152  I69153  I69154  I69159  I69161  I69162  I69163  I69164  I69165  I69169  I69190  I69191  I69192  I69193  I69198  I6920  I69210  I69211  I69212  I69213  I69214  I69215  I69218  I69219  I69220  I69221  I69222  I69223  I69228  I69231  I69232  I69233  I69234  I69239  I69241  I69242  I69243  I69244  I69249  I69251  I69252  I69253  I69254  I69259  I69261  I69262  I69263  I69264  I69265  I69269  I69290  I69291  I69292  I69293  I69298  I6930  I69310  I69311  I69312  I69313  I69314  I69315  I69318  I69319  I69320  I69321  I69322  I69323  I69328  I69331  I69332  I69333  I69334  I69339  I69341  I69342  I69343  I69344  I69349  I69351  I69352  I69353  I69354  I69359  I69361  I69362  I69363  I69364  I69365  I69369  I69390  I69391  I69392  I69393  I69398  I6980  I69810  I69811  I69812  I69813  I69814  I69815  I69818  I69819  I69820  I69821  I69822  I69823  I69828  I69831  I69832  I69833  I69834  I69839  I69841  I69842  I69843  I69844  I69849  I69851  I69852  I69853  I69854  I69859  I69861  I69862  I69863  I69864  I69865  I69869  I69890  I69891  I69892  I69893  I69898  I6990  I69910  I69911  I69912  I69913  I69914  I69915  I69918  I69919  I69920  I69921  I69922  I69923  I69928  I69931  I69932  I69933  I69934  I69939  I69941  I69942  I69943  I69944  I69949  I69951  I69952  I69953  I69954  I69959  I69961  I69962  I69963  I69964  I69965  I69969  I69990  I69991  I69992  I69993  I69998 |
| Dementia | **ICD-10-CM:**  F0150  F0151  F0280  F0281  F0390  F0391  G300  G301  G308  G309  G3101  G3109  G311  G3183 | **ICD-10-CM:**  F0150  F0151  F0280  F0281  F0390  F0391  G300  G301  G308  G309  G311 |
| Chronic pulmonary disease | **ICD-10-CM:**  I272  I2781  I2782  I2783  I2789  I279  J40  J410  J411  J418  J42  J430  J431  J432  J438  J439  J440  J441  J449  J4520  J4521  J4522  J4530  J4531  J4532  J4540  J4541  J4542  J4550  J4551  J4552  J45901  J45902  J45909  J45990  J45991  J45998  J470  J471  J479  J60  J61  J620  J628  J630  J631  J632  J633  J634  J635  J636  J64  J65  J660  J661  J662  J668  J670  J671  J672  J673  J674  J675  J676  J677  J678  J679  J684  J701  J703 | **ICD-10-CM:**  I279  J40  J410  J411  J418  J42  J430  J431  J432  J438  J439  J440  J441  J449  J4520  J4521  J4522  J4530  J4531  J4532  J4540  J4541  J4542  J4550  J4551  J4552  J45901  J45902  J45909  J45990  J45991  J45998  J470  J471  J479  J60  J61  J620  J628  J630  J631  J632  J633  J634  J635  J636  J64  J65  J660  J661  J662  J668  J670  J671  J672  J673  J674  J675  J676  J677  J678  J679  J684  J701  J703 |
| Rheumatologic disease | **ICD-10-CM:**  M0500  M05011  M05012  M05019  M05021  M05022  M05029  M05031  M05032  M05039  M05041  M05042  M05049  M05051  M05052  M05059  M05061  M05062  M05069  M05071  M05072  M05079  M0509  M0510  M05111  M05112  M05119  M05121  M05122  M05129  M05131  M05132  M05139  M05141  M05142  M05149  M05151  M05152  M05159  M05161  M05162  M05169  M05171  M05172  M05179  M0519  M0520  M05211  M05212  M05219  M05221  M05222  M05229  M05231  M05232  M05239  M05241  M05242  M05249  M05251  M05252  M05259  M05261  M05262  M05269  M05271  M05272  M05279  M0529  M0530  M05311  M05312  M05319  M05321  M05322  M05329  M05331  M05332  M05339  M05341  M05342  M05349  M05351  M05352  M05359  M05361  M05362  M05369  M05371  M05372  M05379  M0539  M0540  M05411  M05412  M05419  M05421  M05422  M05429  M05431  M05432  M05439  M05441  M05442  M05449  M05451  M05452  M05459  M05461  M05462  M05469  M05471  M05472  M05479  M0549  M0550  M05511  M05512  M05519  M05521  M05522  M05529  M05531  M05532  M05539  M05541  M05542  M05549  M05551  M05552  M05559  M05561  M05562  M05569  M05571  M05572  M05579  M0559  M0560  M05611  M05612  M05619  M05621  M05622  M05629  M05631  M05632  M05639  M05641  M05642  M05649  M05651  M05652  M05659  M05661  M05662  M05669  M05671  M05672  M05679  M0569  M0570  M05711  M05712  M05719  M05721  M05722  M05729  M05731  M05732  M05739  M05741  M05742  M05749  M05751  M05752  M05759  M05761  M05762  M05769  M05771  M05772  M05779  M0579  M0580  M05811  M05812  M05819  M05821  M05822  M05829  M05831  M05832  M05839  M05841  M05842  M05849  M05851  M05852  M05859  M05861  M05862  M05869  M05871  M05872  M05879  M0589  M059  M0600  M06011  M06012  M06019  M06021  M06022  M06029  M06031  M06032  M06039  M06041  M06042  M06049  M06051  M06052  M06059  M06061  M06062  M06069  M06071  M06072  M06079  M0608  M0609  M061  M0620  M06211  M06212  M06219  M06221  M06222  M06229  M06231  M06232  M06239  M06241  M06242  M06249  M06251  M06252  M06259  M06261  M06262  M06269  M06271  M06272  M06279  M0628  M0629  M0630  M06311  M06312  M06319  M06321  M06322  M06329  M06331  M06332  M06339  M064  M06341  M06342  M06349  M06351  M06352  M06359  M06361  M06362  M06369  M06371  M06372  M06379  M0638  M0639  M0680  M06811  M06812  M06819  M06821  M06822  M06829  M06831  M06832  M06839  M06841  M06842  M06849  M06851  M06852  M06859  M06861  M06862  M06869  M06871  M06872  M06879  M0688  M0689  M069  M315  M316  M320  M3210  M3211  M3212  M3213  M3214  M3215  M3219  M328  M329  M3300  M3301  M3302  M3303  M3309  M3310  M3311  M3312  M3313  M3319  M3320  M3321  M3322  M3329  M3390  M3391  M3392  M3393  M3399  M340  M341  M342  M3481  M3482  M3483  M3489  M349  M3500  M3501  M3502  M3503  M3504  M3509  M351  M352  M353  M354  M355  M356  M357  M358  M359  M360 | **ICD-10-CM:**  M0500  M05011  M05012  M05019  M05021  M05022  M05029  M05031  M05032  M05039  M05041  M05042  M05049  M05051  M05052  M05059  M05061  M05062  M05069  M05071  M05072  M05079  M0509  M0510  M05111  M05112  M05119  M05121  M05122  M05129  M05131  M05132  M05139  M05141  M05142  M05149  M05151  M05152  M05159  M05161  M05162  M05169  M05171  M05172  M05179  M0519  M0520  M05211  M05212  M05219  M05221  M05222  M05229  M05231  M05232  M05239  M05241  M05242  M05249  M05251  M05252  M05259  M05261  M05262  M05269  M05271  M05272  M05279  M0529  M0530  M05311  M05312  M05319  M05321  M05322  M05329  M05331  M05332  M05339  M05341  M05342  M05349  M05351  M05352  M05359  M05361  M05362  M05369  M05371  M05372  M05379  M0539  M0540  M05411  M05412  M05419  M05421  M05422  M05429  M05431  M05432  M05439  M05441  M05442  M05449  M05451  M05452  M05459  M05461  M05462  M05469  M05471  M05472  M05479  M0549  M0550  M05511  M05512  M05519  M05521  M05522  M05529  M05531  M05532  M05539  M05541  M05542  M05549  M05551  M05552  M05559  M05561  M05562  M05569  M05571  M05572  M05579  M0559  M0560  M05611  M05612  M05619  M05621  M05622  M05629  M05631  M05632  M05639  M05641  M05642  M05649  M05651  M05652  M05659  M05661  M05662  M05669  M05671  M05672  M05679  M0569  M0570  M05711  M05712  M05719  M05721  M05722  M05729  M05731  M05732  M05739  M05741  M05742  M05749  M05751  M05752  M05759  M05761  M05762  M05769  M05771  M05772  M05779  M0579  M0580  M05811  M05812  M05819  M05821  M05822  M05829  M05831  M05832  M05839  M05841  M05842  M05849  M05851  M05852  M05859  M05861  M05862  M05869  M05871  M05872  M05879  M0589  M059  M0600  M06011  M06012  M06019  M06021  M06022  M06029  M06031  M06032  M06039  M06041  M06042  M06049  M06051  M06052  M06059  M06061  M06062  M06069  M06071  M06072  M06079  M0608  M0609  M061  M0620  M06211  M06212  M06219  M06221  M06222  M06229  M06231  M06232  M06239  M06241  M06242  M06249  M06251  M06252  M06259  M06261  M06262  M06269  M06271  M06272  M06279  M0628  M0629  M0630  M06311  M06312  M06319  M06321  M06322  M06329  M06331  M06332  M06339  M064  M06341  M06342  M06349  M06351  M06352  M06359  M06361  M06362  M06369  M06371  M06372  M06379  M0638  M0639  M0680  M06811  M06812  M06819  M06821  M06822  M06829  M06831  M06832  M06839  M06841  M06842  M06849  M06851  M06852  M06859  M06861  M06862  M06869  M06871  M06872  M06879  M0688  M0689  M069  M315  M320  M3210  M3211  M3212  M3213  M3214  M3215  M3219  M328  M329  M3300  M3301  M3302  M3303  M3309  M3310  M3311  M3312  M3313  M3319  M3320  M3321  M3322  M3329  M3390  M3391  M3392  M3393  M3399  M340  M341  M342  M3481  M3482  M3483  M3489  M349  M351  M353  M360 |
| Peptic ulcer disease | **ICD-10-CM:**  K250  K251  K252  K253  K254  K255  K256  K257  K259  K260  K261  K262  K263  K264  K265  K266  K267  K269  K270  K271  K272  K273  K274  K275  K276  K277  K279  K280  K281  K282  K283  K284  K285  K286  K287  K289 | **ICD-10-CM:**  K250  K251  K252  K253  K254  K255  K256  K257  K259  K260  K261  K262  K263  K264  K265  K266  K267  K269  K270  K271  K272  K273  K274  K275  K276  K277  K279  K280  K281  K282  K283  K284  K285  K286  K287  K289 |
| Mild liver disease | **ICD-10-CM:**  B180  B181  B182  B188  B189  K700  K7010  K7011  K702  K7030  K7031  K709  K713  K714  K7150  K717  K730  K731  K732  K738  K739  K740  K741  K742  K743  K744  K745  K7460  K7469  K752  K753  K754  K7581  K7589  K759  K760  K761  K762  K763  K764  K7689  K769  K77 | **ICD-10-CM:**  B180  B181  B182  B188  B189  K700  K7010  K7011  K702  K709  K713  K714  K717  K730  K731  K732  K738  K739  K740  K741  K742  K743  K744  K745  K7460  K7469  K760  K762  K763  K764  K769  Z944 |
| Diabetes without chronic complication | **ICD-10-CM:**  E1010  E1011  E10630  E10638  E10641  E10649  E1065  E1069  E108  E109  E1100  E1101  E1110  E1111  E11630  E11638  E11641  E11649  E1165  E1169  E118  E119  E1300  E1301  E1310  E1311  E13630  E13638  E13641  E13649  E1365  E1369  E138  E139 | **ICD-10-CM:**  E108  E109  E118  E119  E138  E139 |
| Diabetes with chronic complication | **ICD-10-CM:**  E1021  E1022  E1029  E10311  E10319  E10321  E103211  E103212  E103213  E103219  E10329  E103291  E103292  E103293  E103299  E10331  E103311  E103312  E103313  E103319  E10339  E103391  E103392  E103393  E103399  E10341  E103411  E103412  E103413  E103419  E10349  E103491  E103492  E103493  E103499  E10351  E103511  E103512  E103513  E103519  E103521  E103522  E103523  E103529  E103531  E103532  E103533  E103539  E103541  E103542  E103543  E103549  E103551  E103552  E103553  E103559  E10359  E103591  E103592  E103593  E103599  E1036  E1037X1  E1037X2  E1037X3  E1037X9  E1039  E1040  E1041  E1042  E1043  E1044  E1049  E1051  E1052  E1059  E10610  E10618  E10620  E10621  E10622  E10628  E1121  E1122  E1129  E11311  E11319  E11321  E113211  E113212  E113213  E113219  E11329  E113291  E113292  E113293  E113299  E11331  E113311  E113312  E113313  E113319  E11339  E113391  E113392  E113393  E113399  E11341  E113411  E113412  E113413  E113419  E11349  E113491  E113492  E113493  E113499  E11351  E113511  E113512  E113513  E113519  E113521  E113522  E113523  E113529  E113531  E113532  E113533  E113539  E113541  E113542  E113543  E113549  E113551  E113552  E113553  E113559  E11359  E113591  E113592  E113593  E113599  E1136  E1137X1  E1137X2  E1137X3  E1137X9  E1139  E1140  E1141  E1142  E1143  E1144  E1149  E1151  E1152  E1159  E11610  E11618  E11620  E11621  E11622  E11628  E1321  E1322  E1329  E13311  E13319  E133211  E133212  E133213  E133219  E133291  E133292  E133293  E133299  E133311  E133312  E133313  E133319  E133391  E133392  E133393  E133399  E133411  E133412  E133413  E133419  E133491  E133492  E133493  E133499  E133511  E133512  E133513  E133519  E133521  E133522  E133523  E133529  E133531  E133532  E133533  E133539  E133541  E133542  E133543  E133549  E133551  E133552  E133553  E133559  E133591  E133592  E133593  E133599  E1336  E1337X1  E1337X2  E1337X3  E1337X9  E1339  E1340  E1341  E1342  E1343  E1344  E1349  E1351  E1352  E1359  E13610  E13618  E13620  E13621  E13622  E13628 | **ICD-10-CM:**  E1021  E1022  E1029  E10311  E10319  E10321  E103211  E103212  E103213  E103219  E10329  E103291  E103292  E103293  E103299  E10331  E103311  E103312  E103313  E103319  E10339  E103391  E103392  E103393  E103399  E10341  E103411  E103412  E103413  E103419  E10349  E103491  E103492  E103493  E103499  E10351  E103511  E103512  E103513  E103519  E103521  E103522  E103523  E103529  E103531  E103532  E103533  E103539  E103541  E103542  E103543  E103549  E103551  E103552  E103553  E103559  E10359  E103591  E103592  E103593  E103599  E1036  E1037X1  E1037X2  E1037X3  E1037X9  E1039  E1040  E1041  E1042  E1043  E1044  E1049  E1121  E1122  E1129  E11311  E11319  E11321  E113211  E113212  E113213  E113219  E11329  E113291  E113292  E113293  E113299  E11331  E113311  E113312  E113313  E113319  E11339  E113391  E113392  E113393  E113399  E11341  E113411  E113412  E113413  E113419  E11349  E113491  E113492  E113493  E113499  E11351  E113511  E113512  E113513  E113519  E113521  E113522  E113523  E113529  E113531  E113532  E113533  E113539  E113541  E113542  E113543  E113549  E113551  E113552  E113553  E113559  E11359  E113591  E113592  E113593  E113599  E1136  E1137X1  E1137X2  E1137X3  E1137X9  E1139  E1140  E1141  E1142  E1143  E1144  E1149  E1321  E1322  E1329  E13311  E13319  E133211  E133212  E133213  E133219  E133291  E133292  E133293  E133299  E133311  E133312  E133313  E133319  E133391  E133392  E133393  E133399  E133411  E133412  E133413  E133419  E133491  E133492  E133493  E133499  E133511  E133512  E133513  E133519  E133521  E133522  E133523  E133529  E133531  E133532  E133533  E133539  E133541  E133542  E133543  E133549  E133551  E133552  E133553  E133559  E133591  E133592  E133593  E133599  E1336  E1337X1  E1337X2  E1337X3  E1337X9  E1339  E1340  E1341  E1342  E1343  E1344  E1349 |
| Hemiplegia or paraplegia | **ICD-10-CM:**  G041  G114  G800  G801  G802  G8100  G8101  G8102  G8103  G8104  G8110  G8111  G8112  G8113  G8114  G8190  G8191  G8192  G8193  G8194  G8220  G8221  G8222  G830  G8310  G8311  G8312  G8313  G8314  G8320  G8321  G8322  G8323  G8324  G8330  G8331  G8332  G8333  G8334  G834  G839  G8250  G8251  G8252  G8253  G8254 | **ICD-10-CM:**  G041  G114  G801  G802  G8100  G8101  G8102  G8103  G8104  G8110  G8111  G8112  G8113  G8114  G8190  G8191  G8192  G8193  G8194  G8220  G8221  G8222  G830  G8310  G8311  G8312  G8313  G8314  G8320  G8321  G8322  G8323  G8324  G8330  G8331  G8332  G8333  G8334  G834  G839  G8250  G8251  G8252  G8253  G8254 |
| Renal disease | **ICD-10-CM:**  E0821  E0822  E0829  I120  I129  I130  I1310  I1311  I132  N030  N031  N032  N033  N034  N035  N036  N037  N038  N039  N052  N053  N054  N055  N056  N057  N059  N062  N063  N064  N065  N072  N073  N074  N075  N08  N171  N172  N181  N182  N183  N184  N185  N186  N189  N19  N250  Z4822  Z4901  Z4902  Z4931  Z4932  Z940  Z992 | **ICD-10-CM:**  I120  N032  N033  N034  N035  N036  N037  N052  N053  N054  N055  N056  N057  N181  N182  N183  N184  N185  N186  N189  N19  N250  Z4901  Z4902  Z940  Z992 |
| Any malignancy, including lymphoma and leukemia | **ICD-10-CM:**  C000  C001  C002  C003  C004  C005  C006  C008  C009  C01  C020  C021  C022  C023  C024  C028  C029  C030  C031  C039  C040  C041  C048  C049  C050  C051  C052  C058  C059  C060  C061  C062  C0680  C0689  C069  C07  C080  C081  C089  C090  C091  C098  C099  C100  C101  C102  C103  C104  C108  C109  C110  C111  C112  C113  C118  C119  C12  C130  C131  C132  C138  C139  C140  C142  C148  C153  C154  C155  C158  C159  C160  C161  C162  C163  C164  C165  C166  C168  C169  C170  C171  C172  C173  C178  C179  C180  C181  C182  C183  C184  C185  C186  C187  C188  C189  C19  C20  C210  C211  C212  C218  C220  C221  C222  C223  C224  C227  C228  C229  C23  C240  C241  C248  C249  C250  C251  C252  C253  C254  C257  C258  C259  C260  C261  C269  C300  C301  C310  C311  C312  C313  C318  C319  C320  C321  C322  C323  C328  C329  C33  C3400  C3401  C3402  C3410  C3411  C3412  C342  C3430  C3431  C3432  C3480  C3481  C3482  C3490  C3491  C3492  C37  C380  C381  C382  C383  C384  C388  C390  C399  C4000  C4001  C4002  C4010  C4011  C4012  C4020  C4021  C4022  C4030  C4031  C4032  C4080  C4081  C4082  C4090  C4091  C4092  C410  C411  C412  C413  C414  C419  C430  C4310  C4311  C4312  C4320  C4321  C4322  C4330  C4331  C4339  C434  C4351  C4352  C4359  C4360  C4361  C4362  C4370  C4371  C4372  C438  C439  C450  C451  C452  C457  C459  C460  C461  C462  C463  C464  C4650  C4651  C4652  C467  C469  C470  C4710  C4711  C4712  C4720  C4721  C4722  C473  C474  C475  C476  C478  C479  C480  C481  C482  C488  C490  C4910  C4911  C4912  C4920  C4921  C4922  C493  C494  C495  C496  C498  C499  C49A0  C49A1  C49A2  C49A3  C49A4  C49A5  C49A9  C4A0  C4A10  C4A11  C4A12  C4A20  C4A21  C4A22  C4A30  C4A31  C4A39  C4A4  C4A51  C4A52  C4A59  C4A60  C4A61  C4A62  C4A70  C4A71  C4A72  C4A8  C4A9  C50011  C50012  C50019  C50021  C50022  C50029  C50111  C50112  C50119  C50121  C50122  C50129  C50211  C50212  C50219  C50221  C50222  C50229  C50311  C50312  C50319  C50321  C50322  C50329  C50411  C50412  C50419  C50421  C50422  C50429  C50511  C50512  C50519  C50521  C50522  C50529  C50611  C50612  C50619  C50621  C50622  C50629  C50811  C50812  C50819  C50821  C50822  C50829  C50911  C50912  C50919  C50921  C50922  C50929  C510  C511  C512  C518  C519  C52  C530  C531  C538  C539  C540  C541  C542  C543  C548  C549  C55  C561  C562  C569  C5700  C5701  C5702  C5710  C5711  C5712  C5720  C5721  C5722  C573  C574  C577  C578  C579  C58  C600  C601  C602  C608  C609  C61  C6200  C6201  C6202  C6210  C6211  C6212  C6290  C6291  C6292  C6300  C6301  C6302  C6310  C6311  C6312  C632  C637  C638  C639  C641  C642  C649  C651  C652  C659  C661  C662  C669  C670  C671  C672  C673  C674  C675  C676  C677  C678  C679  C680  C681  C688  C689  C6900  C6901  C6902  C6910  C6911  C6912  C6920  C6921  C6922  C6930  C6931  C6932  C6940  C6941  C6942  C6950  C6951  C6952  C6960  C6961  C6962  C6980  C6981  C6982  C6990  C6991  C6992  C700  C701  C709  C710  C711  C712  C713  C714  C715  C716  C717  C718  C719  C720  C721  C7220  C7221  C7222  C7230  C7231  C7232  C7240  C7241  C7242  C7250  C7259  C729  C73  C7400  C7401  C7402  C7410  C7411  C7412  C7490  C7491  C7492  C750  C751  C752  C753  C754  C755  C758  C759  C760  C761  C762  C763  C7640  C7641  C7642  C7650  C7651  C7652  C768  C7A00  C7A010  C7A011  C7A012  C7A019  C7A020  C7A021  C7A022  C7A023  C7A024  C7A025  C7A026  C7A029  C7A090  C7A091  C7A092  C7A093  C7A094  C7A095  C7A096  C7A098  C7A1  C7A8  C801  C802  C8100  C8101  C8102  C8103  C8104  C8105  C8106  C8107  C8108  C8109  C8110  C8111  C8112  C8113  C8114  C8115  C8116  C8117  C8118  C8119  C8120  C8121  C8122  C8123  C8124  C8125  C8126  C8127  C8128  C8129  C8130  C8131  C8132  C8133  C8134  C8135  C8136  C8137  C8138  C8139  C8140  C8141  C8142  C8143  C8144  C8145  C8146  C8147  C8148  C8149  C8170  C8171  C8172  C8173  C8174  C8175  C8176  C8177  C8178  C8179  C8190  C8191  C8192  C8193  C8194  C8195  C8196  C8197  C8198  C8199  C8200  C8201  C8202  C8203  C8204  C8205  C8206  C8207  C8208  C8209  C8210  C8211  C8212  C8213  C8214  C8215  C8216  C8217  C8218  C8219  C8220  C8221  C8222  C8223  C8224  C8225  C8226  C8227  C8228  C8229  C8230  C8231  C8232  C8233  C8234  C8235  C8236  C8237  C8238  C8239  C8240  C8241  C8242  C8243  C8244  C8245  C8246  C8247  C8248  C8249  C8250  C8251  C8252  C8253  C8254  C8255  C8256  C8257  C8258  C8259  C8260  C8261  C8262  C8263  C8264  C8265  C8266  C8267  C8268  C8269  C8280  C8281  C8282  C8283  C8284  C8285  C8286  C8287  C8288  C8289  C8290  C8291  C8292  C8293  C8294  C8295  C8296  C8297  C8298  C8299  C8300  C8301  C8302  C8303  C8304  C8305  C8306  C8307  C8308  C8309  C8310  C8311  C8312  C8313  C8314  C8315  C8316  C8317  C8318  C8319  C8330  C8331  C8332  C8333  C8334  C8335  C8336  C8337  C8338  C8339  C8350  C8351  C8352  C8353  C8354  C8355  C8356  C8357  C8358  C8359  C8370  C8371  C8372  C8373  C8374  C8375  C8376  C8377  C8378  C8379  C8380  C8381  C8382  C8383  C8384  C8385  C8386  C8387  C8388  C8389  C8390  C8391  C8392  C8393  C8394  C8395  C8396  C8397  C8398  C8399  C8400  C8401  C8402  C8403  C8404  C8405  C8406  C8407  C8408  C8409  C8410  C8411  C8412  C8413  C8414  C8415  C8416  C8417  C8418  C8419  C8440  C8441  C8442  C8443  C8444  C8445  C8446  C8447  C8448  C8449  C8460  C8461  C8462  C8463  C8464  C8465  C8466  C8467  C8468  C8469  C8470  C8471  C8472  C8473  C8474  C8475  C8476  C8477  C8478  C8479  C8490  C8491  C8492  C8493  C8494  C8495  C8496  C8497  C8498  C8499  C84A0  C84A1  C84A2  C84A3  C84A4  C84A5  C84A6  C84A7  C84A8  C84A9  C84Z0  C84Z1  C84Z2  C84Z3  C84Z4  C84Z5  C84Z6  C84Z7  C84Z8  C84Z9  C8510  C8511  C8512  C8513  C8514  C8515  C8516  C8517  C8518  C8519  C8520  C8521  C8522  C8523  C8524  C8525  C8526  C8527  C8528  C8529  C8580  C8581  C8582  C8583  C8584  C8585  C8586  C8587  C8588  C8589  C8590  C8591  C8592  C8593  C8594  C8595  C8596  C8597  C8598  C8599  C860  C861  C862  C863  C864  C865  C866  C880  C882  C883  C884  C888  C889  C9000  C9001  C9002  C9010  C9011  C9012  C9020  C9021  C9022  C9030  C9031  C9032  C9100  C9101  C9102  C9110  C9111  C9112  C9130  C9131  C9132  C9140  C9141  C9142  C9150  C9151  C9152  C9160  C9161  C9162  C9190  C9191  C9192  C91A0  C91A1  C91A2  C91Z0  C91Z1  C91Z2  C9200  C9201  C9202  C9210  C9211  C9212  C9220  C9221  C9222  C9230  C9231  C9232  C9240  C9241  C9242  C9250  C9251  C9252  C9260  C9261  C9262  C9290  C9291  C9292  C92A0  C92A1  C92A2  C92Z0  C92Z1  C92Z2  C9300  C9301  C9302  C9310  C9311  C9312  C9330  C9331  C9332  C9390  C9391  C9392  C93Z0  C93Z1  C93Z2  C9400  C9401  C9402  C9420  C9421  C9422  C9430  C9431  C9432  C9440  C9441  C9442  C946  C9480  C9481  C9482  C9500  C9501  C9502  C9510  C9511  C9512  C9590  C9591  C9592  C960  C962  C964  C965  C966  C969  C96A  C96Z  D45 | **ICD-10-CM:**  C000  C001  C002  C003  C004  C005  C006  C008  C009  C01  C020  C021  C022  C023  C024  C028  C029  C030  C031  C039  C040  C041  C048  C049  C050  C051  C052  C058  C059  C060  C061  C062  C0680  C0689  C069  C07  C080  C081  C089  C090  C091  C098  C099  C100  C101  C102  C103  C104  C108  C109  C110  C111  C112  C113  C118  C119  C12  C130  C131  C132  C138  C139  C140  C142  C148  C153  C154  C155  C158  C159  C160  C161  C162  C163  C164  C165  C166  C168  C169  C170  C171  C172  C173  C178  C179  C180  C181  C182  C183  C184  C185  C186  C187  C188  C189  C19  C20  C210  C211  C212  C218  C220  C221  C222  C223  C224  C227  C228  C229  C23  C240  C241  C248  C249  C250  C251  C252  C253  C254  C257  C258  C259  C260  C261  C269  C300  C301  C310  C311  C312  C313  C318  C319  C320  C321  C322  C323  C328  C329  C33  C3400  C3401  C3402  C3410  C3411  C3412  C342  C3430  C3431  C3432  C3480  C3481  C3482  C3490  C3491  C3492  C37  C380  C381  C382  C383  C384  C388  C390  C399  C4000  C4001  C4002  C4010  C4011  C4012  C4020  C4021  C4022  C4030  C4031  C4032  C4080  C4081  C4082  C4090  C4091  C4092  C410  C411  C412  C413  C414  C419  C430  C4310  C4311  C4312  C4320  C4321  C4322  C4330  C4331  C4339  C434  C4351  C4352  C4359  C4360  C4361  C4362  C4370  C4371  C4372  C438  C439  C450  C451  C452  C457  C459  C460  C461  C462  C463  C464  C4650  C4651  C4652  C467  C469  C470  C4710  C4711  C4712  C4720  C4721  C4722  C473  C474  C475  C476  C478  C479  C480  C481  C482  C488  C490  C4910  C4911  C4912  C4920  C4921  C4922  C493  C494  C495  C496  C498  C499  C49A0  C49A1  C49A2  C49A3  C49A4  C49A5  C49A9  C50011  C50012  C50019  C50021  C50022  C50029  C50111  C50112  C50119  C50121  C50122  C50129  C50211  C50212  C50219  C50221  C50222  C50229  C50311  C50312  C50319  C50321  C50322  C50329  C50411  C50412  C50419  C50421  C50422  C50429  C50511  C50512  C50519  C50521  C50522  C50529  C50611  C50612  C50619  C50621  C50622  C50629  C50811  C50812  C50819  C50821  C50822  C50829  C50911  C50912  C50919  C50921  C50922  C50929  C510  C511  C512  C518  C519  C52  C530  C531  C538  C539  C540  C541  C542  C543  C548  C549  C55  C561  C562  C569  C5700  C5701  C5702  C5710  C5711  C5712  C5720  C5721  C5722  C573  C574  C577  C578  C579  C58  C600  C601  C602  C608  C609  C61  C6200  C6201  C6202  C6210  C6211  C6212  C6290  C6291  C6292  C6300  C6301  C6302  C6310  C6311  C6312  C632  C637  C638  C639  C641  C642  C649  C651  C652  C659  C661  C662  C669  C670  C671  C672  C673  C674  C675  C676  C677  C678  C679  C680  C681  C688  C689  C6900  C6901  C6902  C6910  C6911  C6912  C6920  C6921  C6922  C6930  C6931  C6932  C6940  C6941  C6942  C6950  C6951  C6952  C6960  C6961  C6962  C6980  C6981  C6982  C6990  C6991  C6992  C700  C701  C709  C710  C711  C712  C713  C714  C715  C716  C717  C718  C719  C720  C721  C7220  C7221  C7222  C7230  C7231  C7232  C7240  C7241  C7242  C7250  C7259  C729  C73  C7400  C7401  C7402  C7410  C7411  C7412  C7490  C7491  C7492  C750  C751  C752  C753  C754  C755  C758  C759  C760  C761  C762  C763  C7640  C7641  C7642  C7650  C7651  C7652  C768  C8100  C8101  C8102  C8103  C8104  C8105  C8106  C8107  C8108  C8109  C8110  C8111  C8112  C8113  C8114  C8115  C8116  C8117  C8118  C8119  C8120  C8121  C8122  C8123  C8124  C8125  C8126  C8127  C8128  C8129  C8130  C8131  C8132  C8133  C8134  C8135  C8136  C8137  C8138  C8139  C8140  C8141  C8142  C8143  C8144  C8145  C8146  C8147  C8148  C8149  C8170  C8171  C8172  C8173  C8174  C8175  C8176  C8177  C8178  C8179  C8190  C8191  C8192  C8193  C8194  C8195  C8196  C8197  C8198  C8199  C8200  C8201  C8202  C8203  C8204  C8205  C8206  C8207  C8208  C8209  C8210  C8211  C8212  C8213  C8214  C8215  C8216  C8217  C8218  C8219  C8220  C8221  C8222  C8223  C8224  C8225  C8226  C8227  C8228  C8229  C8230  C8231  C8232  C8233  C8234  C8235  C8236  C8237  C8238  C8239  C8240  C8241  C8242  C8243  C8244  C8245  C8246  C8247  C8248  C8249  C8250  C8251  C8252  C8253  C8254  C8255  C8256  C8257  C8258  C8259  C8260  C8261  C8262  C8263  C8264  C8265  C8266  C8267  C8268  C8269  C8280  C8281  C8282  C8283  C8284  C8285  C8286  C8287  C8288  C8289  C8290  C8291  C8292  C8293  C8294  C8295  C8296  C8297  C8298  C8299  C8300  C8301  C8302  C8303  C8304  C8305  C8306  C8307  C8308  C8309  C8310  C8311  C8312  C8313  C8314  C8315  C8316  C8317  C8318  C8319  C8330  C8331  C8332  C8333  C8334  C8335  C8336  C8337  C8338  C8339  C8350  C8351  C8352  C8353  C8354  C8355  C8356  C8357  C8358  C8359  C8370  C8371  C8372  C8373  C8374  C8375  C8376  C8377  C8378  C8379  C8380  C8381  C8382  C8383  C8384  C8385  C8386  C8387  C8388  C8389  C8390  C8391  C8392  C8393  C8394  C8395  C8396  C8397  C8398  C8399  C8400  C8401  C8402  C8403  C8404  C8405  C8406  C8407  C8408  C8409  C8410  C8411  C8412  C8413  C8414  C8415  C8416  C8417  C8418  C8419  C8440  C8441  C8442  C8443  C8444  C8445  C8446  C8447  C8448  C8449  C8460  C8461  C8462  C8463  C8464  C8465  C8466  C8467  C8468  C8469  C8470  C8471  C8472  C8473  C8474  C8475  C8476  C8477  C8478  C8479  C8490  C8491  C8492  C8493  C8494  C8495  C8496  C8497  C8498  C8499  C84A0  C84A1  C84A2  C84A3  C84A4  C84A5  C84A6  C84A7  C84A8  C84A9  C84Z0  C84Z1  C84Z2  C84Z3  C84Z4  C84Z5  C84Z6  C84Z7  C84Z8  C84Z9  C8510  C8511  C8512  C8513  C8514  C8515  C8516  C8517  C8518  C8519  C8520  C8521  C8522  C8523  C8524  C8525  C8526  C8527  C8528  C8529  C8580  C8581  C8582  C8583  C8584  C8585  C8586  C8587  C8588  C8589  C8590  C8591  C8592  C8593  C8594  C8595  C8596  C8597  C8598  C8599  C860  C861  C862  C863  C864  C865  C866  C880  C882  C883  C884  C888  C889  C9000  C9001  C9002  C9010  C9011  C9012  C9020  C9021  C9022  C9030  C9031  C9032  C9100  C9101  C9102  C9110  C9111  C9112  C9130  C9131  C9132  C9140  C9141  C9142  C9150  C9151  C9152  C9160  C9161  C9162  C9190  C9191  C9192  C91A0  C91A1  C91A2  C91Z0  C91Z1  C91Z2  C9200  C9201  C9202  C9210  C9211  C9212  C9220  C9221  C9222  C9230  C9231  C9232  C9240  C9241  C9242  C9250  C9251  C9252  C9260  C9261  C9262  C9290  C9291  C9292  C92A0  C92A1  C92A2  C92Z0  C92Z1  C92Z2  C9300  C9301  C9302  C9310  C9311  C9312  C9330  C9331  C9332  C9390  C9391  C9392  C93Z0  C93Z1  C93Z2  C9400  C9401  C9402  C9420  C9421  C9422  C9430  C9431  C9432  C9440  C9441  C9442  C946  C9480  C9481  C9482  C9500  C9501  C9502  C9510  C9511  C9512  C9590  C9591  C9592  C960  C962  C964  C965  C966  C969  C96A  C96Z |
| Moderate or severe liver disease | **ICD-10-CM:**  I8510  I8511  I864  K7040  K7041  K7110  K7111  K7151  K7210  K7211  K7290  K7291  K765  K766  K767  K7681 | **ICD-10-CM:**  I864  K765  K766  K767 |
| Metastatic solid tumor | **ICD-10-CM:**  C770  C771  C772  C773  C774  C775  C778  C779  C7800  C7801  C7802  C781  C782  C7830  C7839  C784  C785  C786  C787  C7880  C7889  C7900  C7901  C7902  C7910  C7911  C7919  C792  C7931  C7932  C7940  C7949  C7951  C7952  C7960  C7961  C7962  C7970  C7971  C7972  C7981  C7982  C7989  C799  C7B00  C7B01  C7B02  C7B03  C7B04  C7B09  C7B1  C7B8  C800 | **ICD-10-CM:**  C770  C771  C772  C773  C774  C775  C778  C779  C7800  C7801  C7802  C781  C782  C7830  C7839  C784  C785  C786  C787  C7880  C7889  C7900  C7901  C7902  C7910  C7911  C7919  C792  C7931  C7932  C7940  C7949  C7951  C7952  C7960  C7961  C7962  C7970  C7971  C7972  C7981  C7982  C7989  C799  C800  C801  C802 |
| AIDS/HIV | **ICD-10-CM:**  B20  B9735  O98711  O98712  O98713  O98719  O9872  O9873  Z21 | **ICD-10-CM:**  B20 |

Note: ICD-10-CM codes typically include a decimal after the first 3 characters. For programming purposes in this dataset, we remove the decimal.

Abbreviations: AIDS/HIV= acquired immunodeficiency syndrome/human immunodeficiency virus; CCI = Charlson comorbidity index, ICD-10 = International Classification of Diseases, Tenth Revision, ICD-10-CM =International Classification of Diseases, Tenth Revision, Clinical Modification, ICD-10-PCS = International Classification of Diseases, Tenth Revision, Procedure Coding System

^a^ Quan et al. Med Care 2005;43:1130-9
